# Carbon‐Based Sunlight Absorbers in Solar‐Driven Steam Generation Devices

**DOI:** 10.1002/gch2.201700094

**Published:** 2018-01-15

**Authors:** Van‐Duong Dao, Ho‐Suk Choi

**Affiliations:** ^1^ Department of Chemical Engineering and Applied Chemistry Chungnam National University 99 Daehak‐ro Yuseong‐Gu Daejeon 305‐764 Republic of Korea; ^2^ Theoretical Physics Research Group Advanced Institute of Materials Science Ton Duc Thang University Ho Chi Minh City Vietnam; ^3^ Faculty of Applied Sciences Ton Duc Thang University Ho Chi Minh City Vietnam

**Keywords:** absorbers, carbon, solar steam generation, solar thermal conversion efficiency

## Abstract

Carbon‐based sunlight absorbers in solar‐driven steam generation have recently attracted much attention due to the possibility of huge applications of low‐cost steam for medical sterilization or sanitization, seawater desalination, chemical distillation, and water purification. In this minireview, recent developments in carbon‐based sunlight absorbers in solar‐driven steam generation systems are reviewed, including graphene, graphite, carbon nanotubes, other carbon materials, and carbon‐based composite materials, highlighting important contributions worldwide that promise low‐cost, efficient, robust, reusable, chemically stable, and excellent broadband solar absorption. Furthermore, the crucial challenges associated with employing carbon materials in this field are emphasized.

## 1 Introduction

Solar energy is well known to be a promising energy source in many applications such as the production of hydrogen,^1^ generation of power,^2^ photovoltaic cells,^3^, ^4^ photocatalysis,^5^, ^6^, ^7^ water purification,^8^ and water desalination.^9^ Among the available applications for solar energy utilization, solar‐driven steam generation (SSG) is one of the most promising applications and it has attracted tremendous attention owing to the wide range of applications.^10^, ^11^, ^12^, ^13^, ^14^, ^15^, ^16^, ^17^, ^18^ The generated steam can be used as a source to produce electricity for worldwide electricity generation,^11^, ^12^, ^13^, ^14^, ^15^ for medical sterilization or sanitization,^16^ and for fresh water production.^17^, ^18^

In order to obtain high efficiency (η) in the performance of SSG, four main factors should be considered; broadband sunlight absorbability, thermal management, water transportation, and water evaporation.^19^, ^20^, ^21^, ^22^, ^23^, ^24^, ^25^, ^26^, ^27^, ^28^, ^29^, ^30^, ^31^, ^32^ Among them, the development of sunlight absorbers with rational structure designs has become a popular research topic, because it has remarkable potential to improve η. The absorbers include metallic plasmonics, semiconductors, carbon, and natural materials.^19^, ^20^, ^21^, ^22^, ^23^, ^24^, ^25^, ^26^, ^27^, ^28^, ^29^, ^30^, ^31^, ^32^ Among absorber materials, carbon‐based materials have low cost, reusability, and excellent light‐to‐heat conversion properties and accordingly have become a common material in our daily life.^26^ However, the operational principle of a carbon‐based sunlight absorber in SSG devices is still not understood. Note that the concept for producing steam is very important to design new materials with enhanced efficiency and stability.

Here we review some recent progress made in the development of carbon‐based sunlight absorbers, including graphene, graphite, carbon nanotubes, other carbon materials, and carbon‐based composites, in SSG regarding their fabrication and characterization with special attention paid to material design. The advantages and disadvantages of carbon‐based absorbers in SSG are highlighted. Moreover, the challenges of high‐performance carbon‐based absorbers in solar‐driven steam are also discussed.

## 2 Steam Generation Mechanism for Carbon‐Based Sunlight Absorbers and Conversion Efficiency Calculation

### 2.1 Steam Generation Mechanism for Carbon‐Based Sunlight Absorbers

The mechanism of a plasmonic SSG system has been explored with two plasmonic models.^10^, ^33^, ^34^, ^35^ The first model is surface plasmon polaritons. In this model, the plasmons are able to travel across the surface in the *x–y* plane of the metal–dielectric interface for tens or hundreds of micrometers and decay to a depth of about 200 nm.^10^, ^33^ The second model is localized surface plasmons. It correlates with the Durde–Lorentz model in solid‐state physics. According to the conduction band, electrons in the metal can be treated as free electrons, but the positive ions are located in fixed positions.^33^

**Figure 1:**
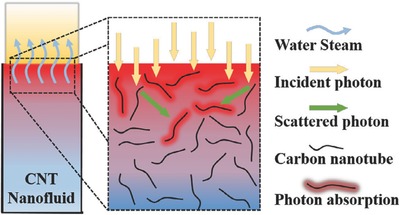
Scheme of localized solar heating and steam‐generation mechanism. Reproduced with permission.^29^ Copyright 2016, Elsevier.

Wang et al. suggested a mechanism for low‐temperature solar vapor generation with carbon nanotubes (CNTs) in a suspended system.^29^ As shown in **Figure** **1**, under solar irradiation, CNTs absorb and scatter photons at the upper fluid first and then a part of photons is incident to the lower fluid. Due to the strong interaction between CNTs and incident solar light, the generation of heat occurs from the surface of the CNTs where strong coupling occurs between the incident radiation and the electrons on the surface of the CNTs.^36^ The generated heat on the surface of the CNTs is then transferred to the media molecular fluids. This generated heat is controlled by the phonon–phonon coupling within the CNTs.^37^ CNTs could enter an excited state with high‐frequency phonon modes. This energy must first be transferred to the low‐frequency modes through phonon–phonon coupling before it can be exchanged with the surrounding medium. This implies that the interface conductance does not depend critically on the surfactant as long as the surfactant is not covalently bonded to the nanotube.^38^ Finally, the thermal energy is diffused in the water.^39^ Therefore, the CNTs near the water–air interface can concentrate light energy to small volumes, resulting in strong localized heating at this area and efficient steam generating from the surrounding liquid.

### 2.2 Conversion Efficiency Calculation

The performance of SSG is estimated through η at steady‐state conditions. The η was provided by Chen and his co‐workers.^40^ They state that it can be calculated by dividing the evaporative heat losses in the generated steam by the total energy for the sample provided by the solar‐radiation input

$$\eta \;\;=\;\;\frac{\dot{m}h_{\mathrm{fg}}}{Q_{s}A}$$

where $\dot{m}$ represents the evaporation flux of water (kg m^−2^ h^−1^), *h*_fg_ is the heat of fusion of vaporization for water (2.257 MJ kg^−1^ at 1 atm for water), *Q_s_* is the power density of light illumination (kW m^−2^), and *A* is the area of the aperture.

## 3 General Design Consideration of Materials for SSG

**Figure 2:**
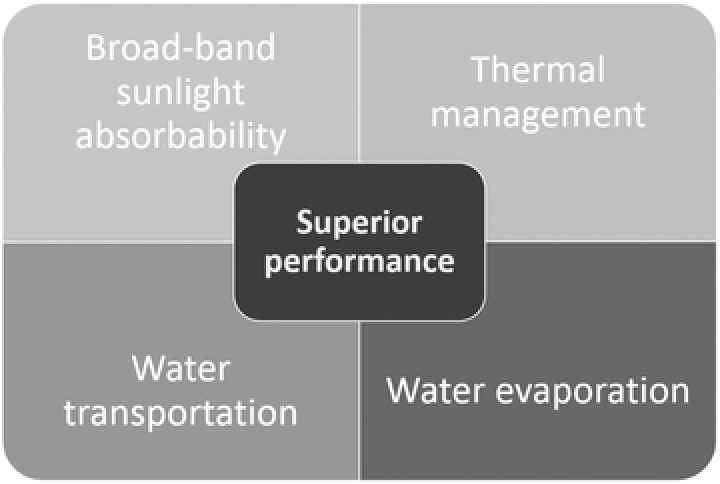
Properties required for high‐performance photothermal materials.

Generally, there are four main factors that affect the conversion efficiency of solar steam generation,^20^, ^21^, ^39^ as shown in **Figure** **2**. The first kind is the broadband sunlight absorbability. The photothermal materials must have a wide absorption capability covering the full solar spectrum range (250–2500 nm). It can be carried out by UV–vis spectrophotometer. The second factor is the thermal management. It is a critical parameter for a heat localization system.^41^ In general, the thermal conductivity is measured by an infrared (IR) microscope. Ghasemi et al. found that low thermal conductivity and limited fluid flow led to the formation of a hot spot and generation of steam at the low optical concentration.^28^ This means the applied materials must be less emissive, which ensures the largest η. The third is the water transportation. It is related to the hydrophilic property or surface property of materials. Finally, the fourth factor is the water evaporation. However, it is not easy for a single material as an absorber to exhibit all factors needed for efficient operation of SSG. In addition, the ideal photothermal materials must be made from world‐abundant elements and can be cost‐effectively scaled‐up for industrial productions.^42^ Furthermore, the other important characteristic of carbon‐based absorbers is its stability which is presented by the adhesion of carbon materials on the substrate during the soaking in water with respect to time. Therefore, the development of low‐cost and scalable photothermal materials that retain light absorption, thermal management, water transportation, and evaporation as well as feasible scalability remains a challenge in relation to current SSG technology.

## 4 Current Developments in Carbon Material Design

**Figure 3:**
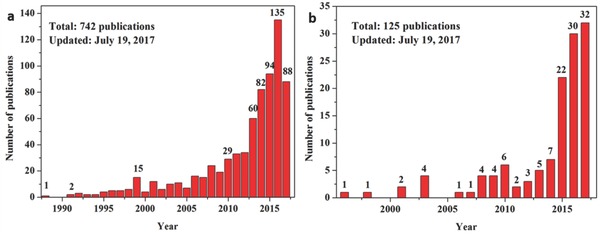
Number of research articles published per year, obtained from a simple and limited literature search using the keywords a) “solar steam generation” and b) “solar steam generation” and “carbon, graphene, carbon nanotube, graphite” (data source: ISI Web of Knowledge).

Carbon materials such as carbon black, graphite, graphene, carbon nanotube, carbon composite, etc. have been tested as a plasmonic solar‐driven steam generation (PSSG) system.^40^ Accordingly, the applied materials act as light‐to‐heat converters and induce rapid water evaporation within just a few seconds upon illumination under different environmental conditions.^10^, ^33^, ^34^, ^35^ Note that this process occurs faster than in conventional SSG systems because of the fast energy release via electron relaxation. However, the lack of in‐depth analysis and a fundamental understanding of the underlying principles of carbon material design can limit the systematic implementation of optimized parameters. Furthermore, as shown in **Figure** **3**, this area is growing fast. A relevant research article is published every three days. An increase in the number of publications results in an abundance of literature in this area. Therefore, communication of the implications of these recent developments in carbon‐based sunlight absorber in SSG devices to the wider scientific community to stimulate further progress is imperative. In this context, we highlighted recent results with the intention of providing information on the design of new carbon‐based photothermal materials in this paper.

**Figure 4:**
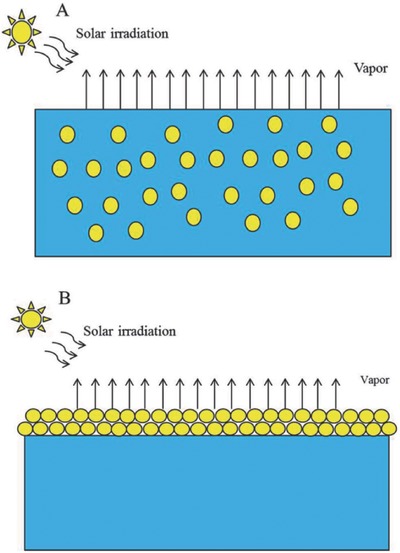
Illustration of plasmonic solar‐driven steam generation systems. a) A suspending system; b) a floating system. Reproduced with permission.^10^ Copyright 2017, Royal Society of Chemistry.

In general, the use of carbon materials in PSSG systems can be classified into two categories (**Figure** **4**).^10^ The first is a suspending system where the applied materials are dispersed in a working fluid. Therefore, it is widely known as a nanofluid,^43^, ^44^, ^45^ or a volumetric system.^40^ The second category is a floating system. In the floating system, the applied materials or composites are assembly floated on the working fluid surface, which is also called an interfacial system. It can be separated by two kinds of floating systems; free‐floating and floating with the help of a supporter.^25^, ^27^, ^46^, ^47^ Actually, the mechanism of steam generation is not similar with different types of PSSG systems. Typical conversion efficiency records of solar steam generation using different carbon materials with different PSSG systems are summarized in **Table** **1**.

**Table 1:** Essential parameters for solar‐driven steam generation system with different carbon materials

Carbon materials	System	Surface chemistry	Light source	Power density [W m^−2^]	Time period [h]	Evaporation rate	Efficiency [%]	Refs.
Flame‐treated wood	Floating system		Solar simulator	1			≈72	48
Hollow carbon beads	Floating system	Hydrophobic	Solar simulator	1	1	1.28 L m^−2^ h^−1^		46
Mushroom	Floating system	hydrophilic	Solar simulator	1			≈62	50
Carbonized mushrooms	Floating system	Hydrophilic	Solar simulator	1			≈78	50
Graphite carbon black	Suspending		Solar simulator	10			67 ± 4	40
Carbon black	Suspending		Solar simulator	10			69 ± 4	40
Graphene	Suspending		Solar simulator	10			68 ± 4	40
Carbon‐black‐based superhydrophobic gauze	Floating system	Hydrophobic	Solar irradiation	1 W cm^−2^	0.5	≈0.1 L h^−1^		49
Carbon‐black‐based superhydrophobic gauze	Floating system	Hydrophobic	Solar irradiation	2 W cm^−2^	0.5	≈0.2 L h^−1^		49
Carbon‐black‐based superhydrophobic gauze	Floating system	Hydrophobic	Solar irradiation	3 W cm^−2^	0.5	≈0.3 L h^−1^		49
Carbon‐black‐based superhydrophobic gauze	Floating system	Hydrophobic	Solar irradiation	5 W cm^−2^	0.5	≈0.5 L h^−1^		49
Carbon‐black‐based superhydrophobic gauze	Floating system	Hydrophobic	solar irradiation	10 W cm^−2^	0.5	≈0.9 L h^−1^		49
Vertically aligned graphene sheets membrane	Floating system		Solar simulator	1		1.62 kg m^−2^ h^−1^	86.50	51
Vertically aligned graphene sheets membrane	Floating system		Solar simulator	4		6.25 kg m^−2^ h^−1^	94.20	51
Chemical reduced graphene oxide	Floating system	Hydrophobic	Solar simulator	1			38.00	55
Exfoliated graphite and carbon foam	Suspending	Hydrophobic	Solar simulator	1			64	28
Exfoliated graphite and carbon foam	Suspending	Hydrophobic	Solar simulator	10			85	28
Nitrogen‐doped porous graphene	Suspending	Hydrophobic	Solar simulator	1	1	1.50 kg m^−2^ h^−1^	80	52
RGO‐sodium alginate carbon nanotube aerogel	Suspending	Hydrophobic	Solar simulator	1	1	1.622 kg m^−2^ h^−1^	83	53
Foldable graphene oxide	Floating system	Hydrophilic	Solar simulator	1			80	25
Graphene aerogel	Floating system	Hydrophilic	Solar simulator	1			53.6 ± 2.5	54
Graphene aerogel	Floating system	Hydrophilic	Solar simulator	10			82.7 ± 2.5	54
Bilayer RGO	Floating system	Hydrophilic	Solar simulator	1		1.31 kg m^−2^ h^−1^	83	54
RGO/mixed cellulose esters	Floating system	Hydrophilic	Solar simulator	1	0.5		60	27
RGO/mixed cellulose esters	Floating system		Solar simulator	4	0.5		71.80	27
Functionalizing graphene	Floating system	Hydrophilic	Solar simulator	1			48	55
Carbon nanotubes	Suspending	Hydrophilic	Solar simulator	10			46.80	29
CNT‐modified flexible wood membrane	Floating system		Solar simulator	10		11.22 kg m^−2^ h^−1^	81	42
CNT/macroporous silica	Floating system	Hydrophobic	Solar simulator	1			82	56
Fe_3_O_4_/RGO	Suspending	Hydrophobic	Solar simulator	1		1.12 L m^−2^ h^−1^	70a)	57
Fe_3_O_4_/C NPs	Floating system		Solar simulator	1.335	5.5	2.3 L m^−2^ h^−1^		58
Fe_3_O_4_/C NPs	Suspending		Solar simulator	1.335	5.5	1.26 L m^−2^ h^−1^		58
GO	Suspending		Solar simulator	0.75			48.40	59
GO + Au 2.6%	Suspending		Solar simulator	0.75			53.80	59
GO + Au 7.8%	Suspending		Solar simulator	0.75			57.40	59
GO + Au 15.6%	Suspending		Solar simulator	0.75			59.20	59
2D graphiyne/CuO‐coated Cu foam	Floating system		Solar simulator	1			91	60
RGO/polyurethane	Floating system		Solar simulator	10			≈81	61
RGO/PS foam	Floating system		Solar simulator	1		1.31 kg m^−2^ h^−1^	83	26
RGO/BNC:BNC aerogel	Floating system	Hydrophilic	Solar simulator	10	300 s	11.8 kg m^−2^ h^−1^		24
3D‐printed porous concave structure	Floating system	Hydrophilic	Solar simulator	1			85.60	21

^a)^ The performance is conducted under water solution contained 3.5% NaCl.

### 4.1 Graphene

Owing to a large surface area, light weight, low molar specific heat, high Debye temperature, outstanding light absorption, and tunable thermal conductivity by chemical doping, graphene is known to be one of promising candidate materials for SSG.^52^

**Figure 5:**
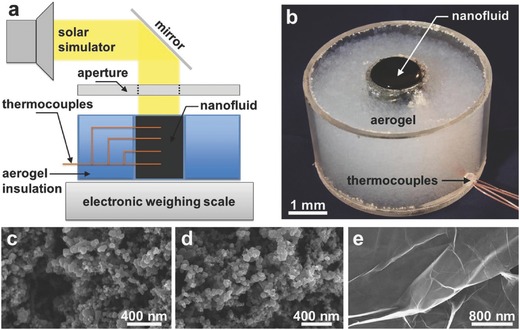
a) Schematic of solar vapor generation device. b) Image of the nanofluid container showing the aerogel insulation, black nanofluid, and thermocouple feed through. Scanning electron micrographs (SEM) of c) graphitized carbon black, d) carbon black, and e) graphene nanoparticles. To obtain SEM images, the nanofluids were dehydrated prior to imaging. Reproduced with permission.^40^ Copyright 2015, Elsevier.

The use of graphene as an absorber in SSG devices can be separated into three routes. The first route is to use graphene in nanofluid systems. In general, a hydrophilic property is required for applied materials. For instance, Chen's group prepared a graphene nanofluid and found 69% conversion efficiency under ten sun illuminations as shown in **Figure** **5**.^40^ Ito et al. presented the fabrication of 3D porous nitrogen‐doped graphene by growth via chemical vapor deposition at different temperatures.^52^ The developed 3D porous nitrogen‐doped graphene materials are applied for SSG devices. As a result, as‐prepared materials have micrometer‐sized pores with an excellent broadband absorption, low heat capacity, and low heat conductivity. The highest η was up to 80% and the steam generation rate was 1.50 kg m^−2^ h^−1^ for nitrogen‐doped porous graphene annealed at a temperature of 950 °C with a 1–2 µm pore size under one sun solar simulation. They also made a comparison in SSG performance between a pore size in nanometers and a pore size of micrometers. As a result, the η of samples with a pore size of micrometers was better than that of samples with a pore size of nanometers. This may be associated with the viscosity of water suppressed in nanosized pore channels, resulting in sluggish delivery of water to hot regions. There are two aspects of nitrogen doping on steam generation. The first one is its lower thermal conductivity and smaller specific heat. And the second aspect is the changing wettability of graphene. The obtained results indicate that there is still space to further improve the steam generation performance of porous graphene by optimizing specific heat, light absorption, thermal conductivity, and water capillary through morphology design and physical property tuning.

**Figure 6:**
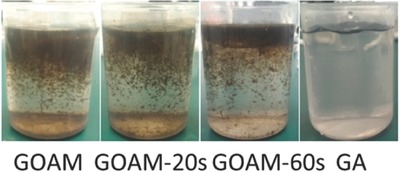
Morphologies of graphene oxide aerogel membranes (GOAMs) in water with different irradiation times after photothermal test. Reproduced with permission.^54^ Copyright 2017, American Chemical Society.

The second approach is to perform the SSG in a floating system. As mentioned above, there are two kinds of floating systems; free‐floating through functional graphene and floating by using Supporting Information. In the case of self‐floating, a hydrophobic property and low density are required for applied materials. For example, Fu et al. used photoreduction of graphene oxide (GO) toward fabricating a graphene aerogel (GA).^54^ The advantage of GA is not only that it converts almost the entire incident solar light to heat energy, but it can also self‐float on the surface of the water and pump the interface water forming a constant vapor, as shown in **Figure** **6**. The conversion efficiencies were found to be 53.6 ± 2.5% and 82.7 ± 2.5% at light intensities of 1 and 10 kW m^−2^, respectively. Wang et al. prepared GO‐based composites on mixed cellulose esters (MCE) with polyethyleneimine as a crosslinking agent.^27^ The reduction of GO to reduced graphene oxide (RGO) was carried out by L‐ascorbic acid. The composite can float on the water–air interface to allow a continuous water supply through the micropores of MCE. Therefore, local heating by minimizing the thermal energy loss for heating the bulk water enabled efficient evaporation of ≈60% under only one sun and 71.8% ± 3% under four sun irradiations. They also found that excellent mechanical stability, low cost, simple preparation, and reusability of the RGO/MCE membrane system can exhibit stable performance over 15 cycles during a cycling test under the same illumination conditions. It has a wide range of practical applications in a large‐scale SSG, sterilization of waste, and seawater desalination.

**Figure 7:**
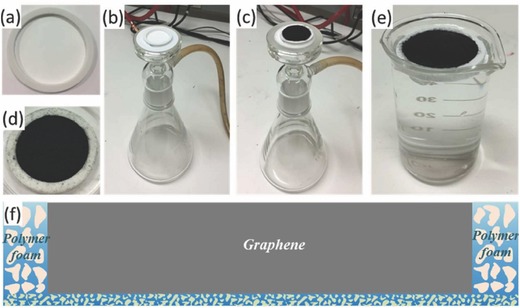
a) The solar absorber cell is made of a polymer ring attached to a hydrophilic polymer membrane. b,c) Graphene derivatives are loaded into the cell by filtering the corresponding dispersions using a vacuum filter. d) The obtained solar‐vapor generator for steam generation. e) This generator is able to float on the water surface and fit snuggly in the beaker. f) A schematic cross‐sectional view of the structure of the generator. Reproduced with permission.^51^ Copyright 2017, American Chemical Society.

In the case of floating with the use of Supporting Information, wood is generally used for this task because it can serve as a thermal insulator to confine the photothermal heat to the evaporative surface and to facilitate the efficient transport of water from the bulk to the photothermally active space. For instance, Liu et al. deposited a GO layer on the microporous wood to provide broad optical absorption and high photothermal conversion, resulting in a rapid increase in the temperature at the liquid surface.^47^ As a result, the wood–GO composite structure provided ≈83% efficiency under simulated solar excitation at a power density of 12 kW m^−2^. Besides wood, the polymer membrane is also used. Zhang et al. prepared a long‐range vertically aligned graphene sheets membrane (VA‐GSM) as a highly efficient solar thermal converter for the generation of clean water. The fabrication procedure is presented in **Figure** **7**. As a result, the average water evaporation rates of 1.62 and 6.25 kg m^−2^ h^−1^ under one and four sun illuminations with a superb η of up to 86.5% and 94.2%, respectively, were achieved for VA‐GSM. As reported, the obtained result is best among carbon materials reported previously. Furthermore, the developed VA‐GSMs can efficiently produce the clean water from seawater, common wastewater, and even concentrated acid and/or alkali solutions.^51^

**Figure 8:**
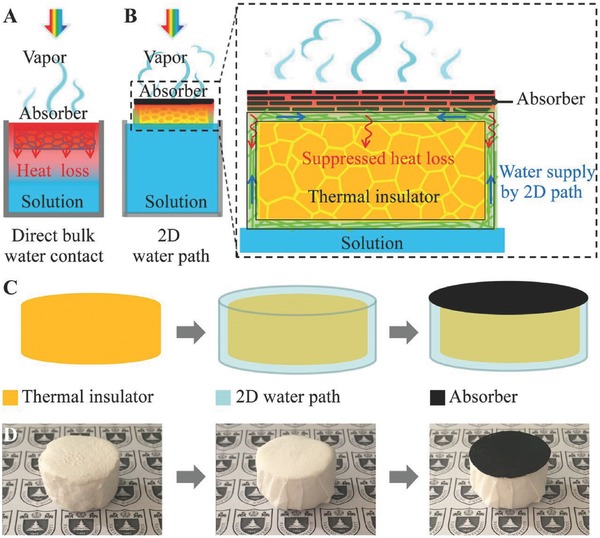
Schematics and the process flow. a) Schematics of a conventional solar steam generation with direct water contact. b) Schematics of solar desalination devices with suppressed heat loss and 2D water supply. c) Flow chart for the fabrication of solar desalination devices: polystyrene foam, cellulose coating, and GO film on the top surface. d) (Left) The physical map of polystyrene foam (thermal insulator), (Middle) cellulose (2D water path) wrapped over the surface of polystyrene foam, and (Right) GO film (absorber) on the top surface. Reproduced with permission.^25^ Copyright 2016, National Academy of Sciences.

The third is to use a thermal insulator to enhance solar steam generation. For this purpose, the hydrophilic property is required for applied materials. Indeed, Yang et al. demonstrated that functionalizing graphene using a hydrophilic group can greatly enhance the solar thermal steam generation efficiency.^59^ They showed that specially functionalized graphene can improve the overall solar‐to‐vapor efficiency from 38% to 48% at one sun condition compared to chemically reduced RGO. Such an improvement is a surface effect that is mainly attributed to the more hydrophilic feature of functionalized graphene, which influences the water meniscus profile at the vapor–liquid interface due to a capillary effect. This will lead to thinner water films close to the three‐phase contact line, where the water surface temperature is higher since the resistance of a thinner water film is smaller, leading to more efficient evaporation. Furthermore, they also reported that this strategy of functionalizing graphene to make it more hydrophilic can be potentially integrated with the existing macroscopic heat isolation strategies to further improve the overall solar‐to‐vapor conversion efficiency. Zhu and co‐workers found simultaneous achievements of an efficient water supply and heat loss suppression by confining the 2D water path.^25^ As a design shown in **Figure** **8**a, a GO absorber is not directly contacted with bulk water because it is separated by a thermal insulator (a polystyrene foam, the thermal conductivity of ≈0.04 Wm K^−1^) to ensure greatly suppressed parasitic heat loss (Figure 8b, zoom‐in). There is a 2D water path on the thin layer of cellulose‐wrapped thermal insulator (Figure 8c,d). The designed 2D water path system presented 80% efficiency under one sun illumination and four orders of magnitude of salinity decrement in a solar desalination device. Interestingly, a foldable GO film becomes an efficient and broadband absorber (>94%) by a standard solar spectrum of AM 1.5 global, suggesting scalable processes and high and stable efficiency under normal solar illumination independent of water quantity without any supporting systems.

### 4.2 Carbon Nanotubes

Besides carbon black, graphene, and graphite, CNTs have also more potential in SSG application. These are generally applied through nanofluids or suspension systems. However, it is well known that the disadvantage of using CNTs in the fabrication of suspension systems is the hydrophobic property of CNTs. Thus, to prepare nanofluids, CNTs should be treated with base media,^63^ or plasma‐functionalized CNTs.^64^ Recently, Wang et al. conducted experiments on direct solar vapor generation enabled by CNT nanofluids.^29^ They discussed the effects of solar‐power density and CNT concentration on SSG performance and showed that an increase in solar power and CNT concentration results in an increased evaporation rate. The η was found to be 46.8% with a concentration of 19.04 × 10^−4^ vol% CNT under ten sun illuminations. More strikingly, they found that the evaporation rate of the localized heating of the nanofluid is higher than that of a bulk temperature increase.

**Figure 9:**
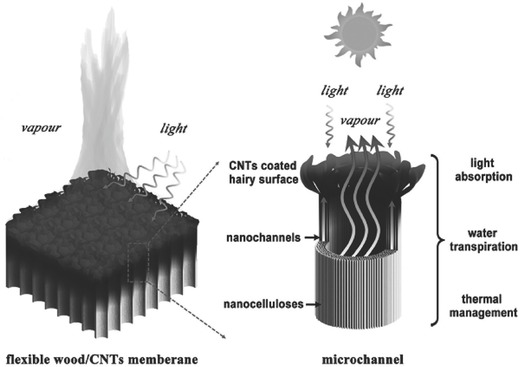
Graphical illustration of flexible solar steam made from a CNT‐coated flexible wood membrane. The F‐Wood/CNTs membrane possesses three advantages as a solar steam: the black hair‐like surface coated with CNTs is highly absorbing to incident sunlight, the microchannels and nanochannels can pump water up, and the wood itself has low thermal conductivity. Reproduced with permission.^42^ Copyright 2017, Wiley‐VCH.

In order to improve the conversion efficiency, reduce costs, and enlarge scale‐up, Chen et al. recently fabricated a CNT‐modified flexible wood membrane, as shown in **Figure** **9**.^42^ Since the wood matrix had excellent light absorbability and low thermal conductivity, the designed materials with hierarchical micro‐ and nanochannels for water pumping and escaping showed a conversion efficiency of 81% with an evaporation rate of 11.22 kg m^−2^ h^−1^ under 10 sun illuminations. An η of 82% under one sun illumination was achieved by Wang's group after optimizing a top self‐floating hydrophobic CNT membrane and a bottom hydrophilic macroporous silica substrate.^56^ They also reported excellent performance of the bilayered material toward water evaporation from seawater and contaminated water, realizing the separation of water from pollutants and indicating its application versatility.

### 4.3 Other Carbon Materials

Owing to their abundant resources, outstanding properties, and environmentally friendly characteristics, carbon materials are ubiquitous and indispensable in present scientific applications.^46^ Carbon‐based nanoparticles (NPs) have better broadband solar absorbance.^40^ Therefore, carbon materials recently have been applied as a low‐cost absorber in SSG devices.^40^, ^46^, ^48^, ^49^, ^50^

**Figure 10:**
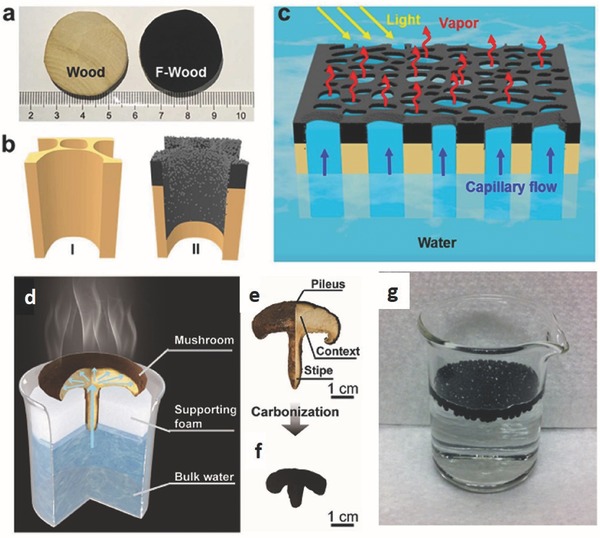
Schematic of F‐wood for a solar steam generation. Reproduced with permission.^48^ Copyright 2017, American Chemical Society. a) Photographs of blocks of pristine wood and F‐wood. The wood blocks were ≈3.2 cm in diameter and 1 cm in thickness. b) Schematics of the inner structures of the I) pristine wood and II) F‐wood. c) Schematic illustration of F‐wood for a solar steam generation. F‐wood floats on the water pump water spontaneously through its inner channels to the heated surface and generates high‐temperature steam for fresh water. Mushroom‐based solar steam generation. Reproduced with permission.^50^ Copyright 2017, Wiley‐VCH. d) Schematic of a mushroom‐based solar steam‐generation device. e) The physical picture of a shiitake mushroom. f) The physical picture of a mushroom after carbonization. (g) Photographic image of carbon beads floated on water surface. Reproduced with permission.^46^ Copyright 2014, Elsevier.

In general, the carbon materials are prepared by carbonization of natural materials such as wood, beads, mushrooms, and so on, as shown in **Figure** **10**. For example, Xue et al. utilized natural wood as a renewable, scalable, low‐cost, and robust material for solar steam applications after a simple flame treatment (Figure 10a–c) because it has very high solar absorbance (≈99%), low thermal conductivity (0.33 W m^−1^ K^−1^), and good hydrophilicity.^48^ The flame‐treated wood can localize the solar heating at the evaporation surface and enable a solar‐thermal efficiency of ≈72% under 1 kW m^−2^ solar intensity. Instead of wood, Zhu and co‐workers found that mushrooms, as a kind of living organism, were efficient SSG devices (Figure 10d–f).^50^ Due to the unique natural structure of mushrooms, including an umbrella‐shaped black pileus, a porous stem, and a fibrous stipe with a small cross‐section, the conversion efficiencies of ≈62% and ≈78% for SSG devices with natural and carbonized mushrooms, respectively, are achieved under one sun illumination. They found that the features of natural mushroom structure not only provide efficient light absorption, water supply, and steam emissions, but also suppress the three components of heat loss at the same time. In the same concept as utilizing the advantages of natural materials such as lightweight, hydrophobicity, and photothermal conversion in near‐infrared regions, Wang and co‐workers used hollow carbon beads that float freely on the surface of the water for steam generation (Figure 10g).^46^ The steam generation rate of the floating system reached 1.28 L m^−2^ h^−1^ under 1 kW m^−2^ solar illumination by using 714 g m^−2^ hollow carbon beads, which was around 237% higher than that of devices without using carbon beads. They also found a relationship between particle density and particle size for the rate of water evaporation. It was also reported that the size of 1.5 mm diameter is suitable for practical use in order to obtain a high evaporation rate with a large amount of particles. With a few number of particles, however, the highest steam generation was obtained at a large size of 3 mm in diameter. This is due to the higher surface effective area of total small particles compared to a small amount of larger particles.

Recently, Liu et al. reported a low‐cost, scalable, and easy‐to‐prepare carbon‐black‐based super‐hydrophobic gauze, which was able to float on the surface of water and selectively heat the surface water under irradiation, resulting in an enhanced evaporation rate.^49^ It was found that the evaporation rate of the floating black gauze was improved by two to three times in comparison with the traditional process due to the higher temperature of surface water in the floating gauze group.

In order to compare the efficiencies of graphite carbon black, carbon black, and graphene, the performance of SSG was evaluated in a suspending SSG system by Chen and co‐workers.^40^ The nanofluids were prepared by sonicating 0.5 wt% of various NPs in distilled water for 1 h. They found that the efficiencies of 67%, 69%, and 68% under ten sun solar illuminations were calculated for graphite carbon black, carbon black, and graphene nanofluids, respectively. The transient experiment confirmed that the system could rapidly respond to a change in the solar illumination angle (Figure 5).

### 4.4 Composite Materials

#### 4.4.1 Carbon/Metal Composite

**Figure 11:**
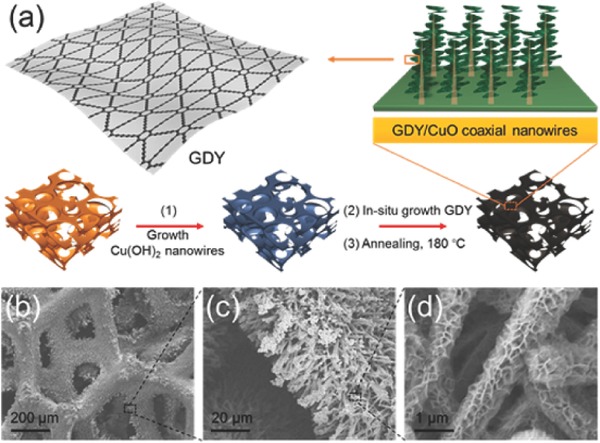
a) Schematic illustration of GDY‐based hierarchical architecture. b–d) SEM images of the copper foam coated by GDY/CuO coaxial nanowires. Reproduced with permission.^60^ Copyright 2017, American Chemical Society.

It is known that metal NPs such as Au, Ag, Cu, etc. have tunable surface plasmon resonance (SPR), resulting in strong capability of tailoring their density of states (DOS), light trapping, and light conversion properties to generate heat at a nanometer length scale by various processes.^64^ As mentioned above, carbon materials have recently been used in SSG devices. Hence, considering all these factors, to obtain broadband absorption in the solar spectrum due to the coupling between the Ag NPs and the RGO, Sharma and Rabinal prepared a graphene–Ag composite after coreduction of GO and Ag^+^ via NaBH_4_ to Ag atoms and RGO, respectively, in a water bath for 5 h.^64^ They found that the absorption and conversion of solar radiation were enhanced with the presence of Ag/RGO composite in water bath.^64^ Mei and co‐workers presented nanocomposites of GO and Au to generate solar vapor under 0.75 sun illumination.^59^ As a result, the conversion efficiency was 53.8%, 57.4%, and 59.2% for nanocomposites of GO with Au 2.6%, Au 7.8%, and Au 15.6%, respectively, which is better than the values of 16.2%, 21.5%, and 48.4% for pure water, Au nanofluids, and GO nanofluids, respectively. Very recently, Gao et al. prepared a new structure based on 3D copper foam supported 1D CuO nanowires anchored with vertical 2D graphiyne (GDY), which has a highly π‐conjugated structure of sp‐ and sp^2^‐hybridized carbon.^60^ The fabrication process of the GDY‐based hierarchical architecture is shown in **Figure** **11**. This architecture has several advantages; (i) the copper foam provides a self‐supporting skeleton; (ii) CuO nanowires are used as an absorber; (iii) GDY nanosheets can simultaneously trap light via structural factors (by increasing the light traveling distance inside materials) and enhance the light absorption through their intrinsic narrow band gap. Thus, this architecture is used as free‐floating foam with a multidimensional architecture, which exhibited highly efficient solar steam generation. As a result, the GDY/CuO copper foam reached 91% efficiency under one sun illumination.^60^

Magnetic Fe_3_O_4_‐based composites were also used in this task. Wu and co‐workers found a conversion efficiency of 70% and a steam generation rate of 1.12 L m^−2^ h^−1^ in a 3.5% NaCl solution, under one sun illumination with an RGO/Fe_3_O_4_ NPs composite.^57^ Zeng et al. fabricated floating Fe_3_O_4_/C magnetic particles with an average size of 500 nm by carbonization of poly(furfuryl alcohol) (PFA) incorporated with Fe_3_O_4_ NPs. The synthesized composite has a hydrophobicity property and a bulk packing density of 0.53 g cm^−3^, and therefore it was floatable on the water. The obtained results indicate that the water evaporation rate was enhanced by as much as a factor of 2.3 in the solar evaporation of 3.5% salt water. They also found that the fabricated composites were easily recycled using a magnet, and stable after being recycled three times.^58^

#### 4.4.2 Carbon/Carbon Composite

**Figure 12:**
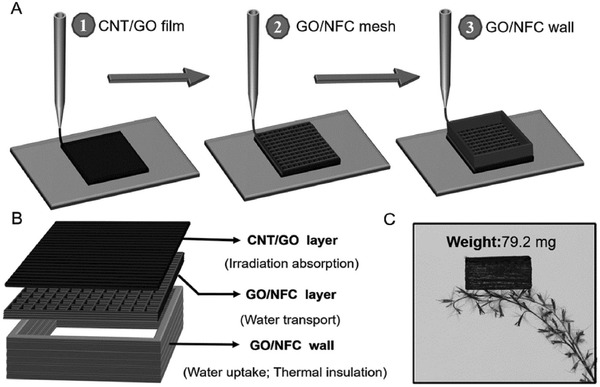
Schematic illustration of the 3D printing process and sample structure. a) Schematic showing the process of 3D printing fabrication. b) Schematic illustration of the structure of the 3D‐printed evaporator, which consists of CNT/GO layer, GO/CNT layer, and GO/NFC wall. c) Photograph demonstrating the light weight of the evaporator. Reproduced with permission.^21^ Copyright 2017, Wiley‐VCH.

Chen and co‐workers employed a double‐layer structure combining exfoliated graphite and carbon foam in a solar steam generation system.^28^ The developed system has four characteristics: solar spectrum absorbability, thermally insulating ability, hydrophilicity, and interconnected porosity. Thus, the conversion efficiency of the system was found to be 64% at one sun and 85% at ten sun solar illuminations. Hu and co‐workers used layer‐by‐layer 3D‐printing to construct an all‐in‐one evaporator for highly‐efficient solar steam generation under one sun illumination.^21^ The 3D‐printed porous concave structure is composed of porous CNT/GO, a GO/nano‐fibrillated cellulose (NFC) layer, and a GO/NFC wall, as shown in **Figure** **12**. The advantage of this structure is efficient broadband solar absorption (>97%) due to the thin CNT/GO layer, and an open porous structure for vapor escape. The connection between the GO/NFC layer and the CNT/GO layer assists water transportation in its channels and supplies water to the evaporation surface. Furthermore, continuous water pathways are formed owing to the porous GO/NFC wall. Thus, the conversion efficiency reached 85.6% under one sun illumination.

Considering the excellent absorption of the solar spectrum, good hydrophilicity, and porous networks for efficient water supply and vapor channels, as well as the thermally insulating property for heat localization, Zhu and co‐workers recently prepared a free‐GO‐based aerogel with a porous structure built up with RGO sheets together with CNTs and sodium alginate (SA).^53^ The fabricated structure exhibited solar conversion efficiency of 83% and the highest steam generation rate (1.622 kg m^−2^ h^−1^) among the samples under one sun solar illumination for 1 h.

#### 4.4.3 Carbon/Polymer Composite

As a result of providing excellent stability, hydrophilic property, broad optical absorption, and thermal insulation, composites of carbon materials with a polymer have been considered as other candidate absorbers in SSG systems. Several types of polymers can be used in this field such as polyurethane (PU),^61^ polystyrene (PS),^26^ polydimethylsiloxane,^65^ polydopamine (PDA),^66^ polypyrrole (PPy),^67^ and poly(acrylonitrile) (PAN).^68^

Wang et al. introduced a novel photoreceiver composed of RGO and a PU matrix to obtain excellent stability and broad optical absorption, together with the property of thermal insulation served by PU resulting in a rapid increase of local temperature under illumination.^61^ Furthermore, the developed structure also presents hydrophilic segments and the interconnected pores of RGO/PU can serve as water channels for replenishment of evaporated surface water. Thus, the conversion efficiency reached ≈81% under ten sun illuminations.

Shi et al. rationally designed and fabricated a bilayered photothermal membrane with a porous film of RGO on the top and PS foam at the bottom.^26^ They carefully optimized the bilayer membrane for SSG. As a result, the highest evaporation rate was 1.31 kg m^−2^ h^−1^ with light to evaporation conversion efficiency of 83% under one sun illumination for use of the optimized bilayered photothermal membrane.

**Figure 13:**
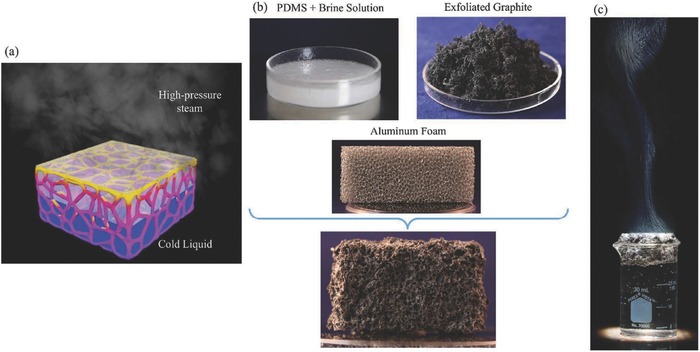
Schematic diagram of an artificially‐networked‐structure and the steam generation experiment. a) Schematic illustration of an artificially networked structure with flexibility for ambient/high‐pressure solar steam generation. b) The components for the development of this structure and the final material structure are shown. The components include polydimethylsiloxane and brine solution, exfoliated graphite, and scarifying aluminum foam. c) The bulk of the fluid is cold and the steam is generated at the surface of this structure at both ambient and high pressures. Reproduced with permission.^65^ Copyright 2016, Royal Society of Chemistry.

Recently, in order to produce high‐pressure steam, an artificially‐networked‐structure with flexibility in a floating PSSG system, which combines polydimethylsiloxane and brine solution, exfoliated graphite, and scarifying aluminum foam, was developed by Ghasemi's group.^65^ Due to the excellent water transportation and low thermal conductivity properties of the 3D artificial material, the steam can be generated at a high pressure of 525 kPa and high‐temperature steam of 156 °C under solar Irradiation (**Figure** **13**).

## 5 Typical Fabrication Techniques

**Figure 14:**
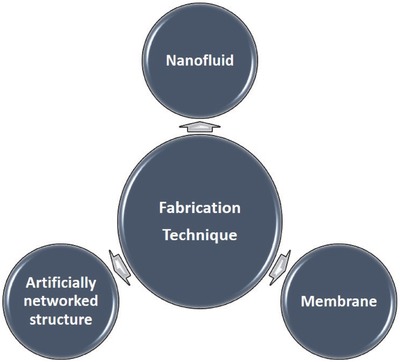
Illustration of typical fabrication techniques in solar‐driven steam generation.

The fabrication of carbon‐based sunlight absorbers in SSG is an important part of absorber development and improvement of η. Typical fabrication techniques are presented in **Figure** **14**.

### 5.1 Fabrication of Nanofluids

Nanofluids are usually applied for suspending systems. In this case, the carbon materials should be well dispersed in the solution. However, it is known that carbon materials generally have a hydrophobic property. Therefore, modification of the carbon materials or the use of surfactants is required for this purpose. In the case of modification, the carbon materials are treated with base media,^69^ the use of plasma,^63^ or the use of a sodium dodecyl sulfate (SDS) surfactant (1 wt%) in solution.^37^ Recently, sonication was also used to fabricate carbon nanofluids^40^ and nanohybrid nanofluids.^59^

### 5.2 Fabrication of Carbon Membrane

In general, a carbon membrane is used for the floating system. There are several routes to fabricate a carbon membrane including vacuum filtration, dipcoating, dropcasting, freezecasting, and printing. Vacuum filtration is a popular method to fabricate carbon membranes. As can be seen in Figure 8, the procedure is very simple and cost‐effective. The carbon membrane can be coated on different substrates such as cellulose^25^ and MCE.^27^ Dipcoating and dropcasting methods are also used. For these methods, the supported substrates are just dropped or dipped in the carbon solution and dried. Wood and air‐laid paper are usually selected for these approaches,^42^, ^47^, ^62^ along with carbon foam^28^ and aluminum foam.^65^

### 5.3 Fabrication of the Artificially Networked Structure

In order to improve the conversion efficiency for SSG, the fabrication of 3D structures is a promising process. For this purpose, the template route is known as an effective method. There are two classifications. The first kind is a soft‐template. In this method, the SBA‐15 powder is used to obtain a uniform mixture of GO solution, which is coated on the PS substrate.^26^ Before the SBA‐15 is removed by treating the bi‐layer membrane with 3M NaOH solution for 8 h, the bilayer membrane is dried in an oven at 60 °C and GO is reduced to RGO by using HI vapor. Thus, the porous RGO membrane is formed. The second is a hard template. The hard template such as aluminum foam can be removed by 35–38% hydrochloric acid (HCl) (Figure 13),^65^ but it also can be kept on an artificially networked structure such as copper foam (Figure 11)^60^ or carbon foam.^28^ In this process, the hard template was impregnated into the developed carbon solution, which is first prepared by a mixture of carbon materials with a binder and thinner. The foam was then dried in the oven. The hard template can then be removed or kept on the final structure up to the used purpose.

**Figure 15:**
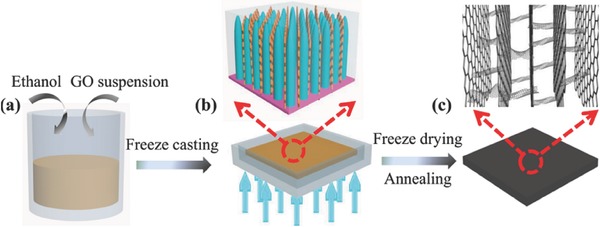
Schematic of the fabrication process and characterization of VA‐GSM. a) GO suspension with a small amount of ethanol. b) Directional freeze casting of GO mixture in a PTFE mold, which is placed on the surface of liquid nitrogen to induce the freezing direction from the bottom to top. c) VA‐GSM is obtained after freeze‐drying and thermal annealing. Reproduced with permission.^51^ Copyright 2017, American Chemical Society.

To obtain artificially networked structures without using any templates, the integrated freeze‐drying and thermal annealing is one of the best routes. The process is a two‐step method. Until now, this route was only applied for fabricating 3D graphene from GO.^51^, ^54^ The first step is freeze‐drying of a GO solution and the second step is the reduction process of GO to RGO by annealing^51^ or irradiation.^54^ As shown in **Figure** **15**, the process starts with the preparation of the mixture of GO with ethanol, and the mixture was transferred into a PTFE mold with a depth of several millimeters.^51^ To obtain directional freeze casting from the bottom to the top, the mold was placed on the surface of liquid nitrogen for 10 min. Finally, the mold was annealed at 200 °C for 1 h and then treated at 1000 °C for 2 h under a nitrogen atmosphere. Instead of annealing, Zhang et al. used ten sun irradiations to reduce GO into RGO.^54^

Recently, printing methods to fabricate 3D structures have also garnered a lot of attention. The fabrication process is illustrated in Figure 12.^21^ The process starts with the preparation of composite inks. 3D printing fabrication is then conducted using a benchtop robot, which is controlled by programmed procedures to print out the designed patterns on a flat ceramic wafer. Thus, this route has very high productivity. After printing, the solvent is removed by transferring the samples into a freeze dryer. Finally, the structure is stabilized through annealing at 140 °C in an argon atmosphere.

## 6 Summary and Outlook

In this minireview, we presented the recent progress and important worldwide contributions of carbon‐based sunlight absorbers in SSG systems such as carbon black, graphene, CNTs, and their composites. A conceptual framework for the design, synthesis, and characterization of carbon‐based absorbers in SSG devices has been discussed and reviewed. Owing to their low cost and high efficiency, carbon materials have remarkable potential for future applications. A GDY‐based free‐floating foam, with a multidimensional architecture that showed excellent solar energy absorption over the whole solar spectrum, and porous networks for efficient vapor flow, reached a record solar‐thermal conversion efficiency of 91% under one sun illumination. Moreover, carbon‐based nanohybrid materials exhibited excellent performance due to the synergetic effects of their different components of nanohybrid materials.

New advances in carbon‐based absorbers for SSG system are required to meet high conversion efficiency, low cost, reusability, chemical stability, and mass production. There are still several steps needed for optimization of conversion efficiency employing a carbon‐based absorber in an SSG system in the future;

(1) As mentioned above, there are four main factors that affect the conversion efficiency of solar steam generation. It is including wide absorption capability, low thermal conductivity, water transportation, and water evaporation. However, it is not easy for a single material as an absorber to exhibit all factors needed for efficient operation of SSG. The development of advanced materials that can satisfy all factors needed for efficient operation of SSG is desired.
(2) CNTs showed excellent performance. However, the effect of length, diameter/wall number, metallic/semiconducting nature, CNTs' functionalization (such as N‐doping, plasma treatment, and so on.), aggregation/distribution, and CNTs' concentration on the conversion efficiency have not been studied or optimized yet. In case of using graphene, the size and layer numbers of graphene should also be considered. Furthermore, the optimization of specific heat, light absorption, thermal conductivity, and water capillary force through morphology design and physical property tuning should also be considered.
(3) Due to the synergistic effect, metal NPs‐loaded carbon materials have received a lot of attention for SSG. Note that the metal NPs have tunable SPR, resulting in the strong capability of tailoring their DOS, light trapping, and light conversion properties to generate heat at a nanometer length scale by various processes.^64^ Thus, the optimum particle size and metal loading on carbon materials should be considered.
(4) Carbon dots (including graphene dots and their functionalized ones) and MXenes (incorporating early transition metal carbides and/or carbonitride) have been steadily advancing into diverse fields such as electrocatalysts, supercapacitors, electromagnetic interference shielding materials, and solar cells, but are still not applied in this field.
(5) The floating system has notable potential to improve conversion efficiency. However, there is a high heating loss through thermal radiation, resulting in low conversion efficiency. Thus, employing converters with low emissivity is required for this purpose.
(6) To date, the mechanism for low‐temperature SSG with carbon‐based absorbers is not well understood. It is still a critical subject that needs to be explored to provide a theoretical basis for designing new advances in carbon‐based absorbers for SSG systems.
(7) The humidity and temperature of a PSSG system also affect η. Lower humidity and a higher temperature can help vapor generation. However, this has not been considered yet.
(8) To date, the PSSG system can only generate vapor under sunlight illumination. This is a limitation for an all‐weather SSG.

## Conflict of Interest

The authors declare no conflict of interest.
